# Diversity on location

**DOI:** 10.7554/eLife.76818

**Published:** 2022-02-07

**Authors:** Young J Yoon, Gary J Bassell

**Affiliations:** 1 Department of Neuroscience, Albert Einstein College of Medicine New York United States; 2 Department of Cell Biology, Emory University School of Medicine Atlanta United States

**Keywords:** FMRP, local translation, mRNA localization, fragile x syndrome, RNA-binding proteins, synaptic plasticity, Mouse

## Abstract

The RNA binding protein FMRP regulates the synthesis of synaptic and nuclear proteins within different compartments of a neuron.

**Related research article** Hale CR, Sawicka K, Mora K, Fak JJ, Kang JJ, Cutrim P, Cialowicz K, Carroll TS, Darnell RB. 2021. FMRP regulates mRNAs encoding distinct functions in the cell body and dendrites of CA1 pyramidal neurons. *eLife* **10**:e71892. doi: 10.7554/eLife.71892

Our fascination with memory dates to antiquity and has not ceased in modern days. While it is relatively straightforward to demonstrate how cognitive associations are formed, the underlying cellular and molecular processes are much harder to pin down.

When the brain forms memories or learns new tasks, the synaptic connections between the neurons are either strengthened or weakened in a process known as plasticity. To remodel synaptic connections and stabilize newly made memories, more proteins must be made: consequently, the way that gene expression is regulated in specific neurons within the hippocampus changes.

Previous research has shown that messenger RNA molecules (mRNAs) are transported to different parts of the neuron, such as the axons and dendrites (the projections of a neuron sending or receiving signals to and from other neurons), whereas others remain in the cell body (Holt et al., 2019). Each localized mRNA acts as a template to make new proteins and thus, local protein synthesis drives plasticity in hippocampal neurons. But exactly how gene expression via mRNA sorting is regulated in the different parts of the neuron has been less clear.

Neurogenetic diseases that lack essential regulators of gene expression may provide clues on how proteins are produced in the correct place, or taken there, during learning and memory. For example, people with Fragile X Syndrome (FXS), a genetic condition that causes intellectual disability and autism, lack the mRNA binding protein FMRP (Santoro et al., 2012). In healthy individuals, FMRP is widely distributed throughout the neuron, where it can regulate the translation of mRNAs to make new proteins and control other aspects of the post-transcriptional process, including the transport and stability of mRNA (Bassell and Warren, 2008). Moreover, FMRP can often repress translation, keeping the mRNA silent until proteins are needed. Upon synaptic stimulation, FMRP’s repressive role is relieved, allowing ribosomes to make new proteins locally.

In FXS, the loss of FMRP causes an overproduction of proteins, which can affect the development and plasticity of synapses (Figure 1; Richter et al., 2015). Since FMRP can travel in and out of the nucleus and to the synapses, it could be an ideal candidate to control the translation of mRNAs into proteins relevant for both nuclear and synaptic roles. However, it has so far been unclear how FMRP could achieve this.

**Figure 1.**
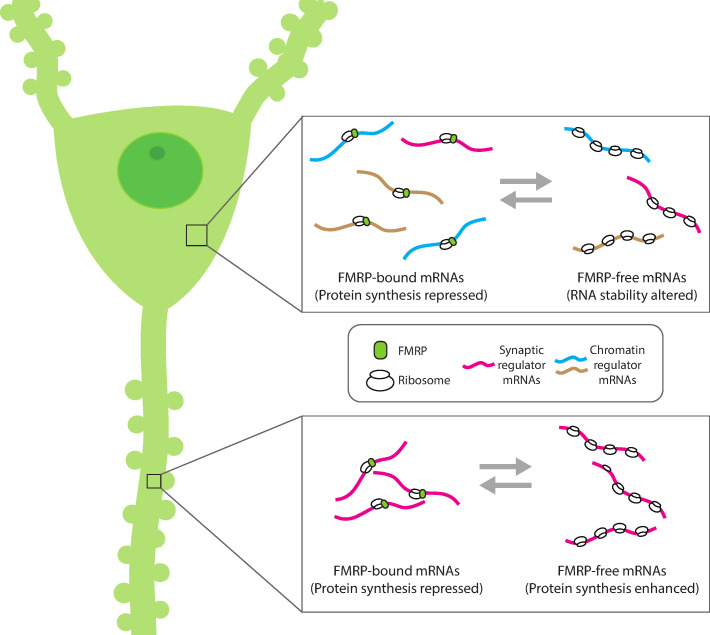
Compartment-specific regulation of mRNA translation by FMRP in neurons. Hale et al. found that the mRNA binding protein FMRP plays an expanded role in regulating protein synthesis in hippocampal CA1 neurons (left). In the cell body (top inset), FMRP (green circle) binds and blocks the translation of synaptic (magenta) and chromatin regulator mRNA (blue and gold). Without FMRP, the stability and/or translation of its target mRNA in the cell body is altered. In the dendrite (bottom inset), FMRP represses the translation of synaptic regulator mRNA, consistent with the role of FMRP to modulate protein synthesis. This suggests that FMRP can interact with different populations of mRNA within subcellular compartments to both reversibly repress translation and regulate the availability of mRNA for protein synthesis.

Now, in eLife, Robert Darnell and colleagues at The Rockefeller University – including Caryn Hale as first author – report that FMRP plays different roles in specific parts of a neuron (Hale et al., 2021). The team created genetically modified mice to analyze mRNAs from the dendrites and the cell bodies of hippocampal neurons involved in memory, known as excitatory CA1 pyramidal neurons. Three variations of genetically modified mice were used in which specific proteins were tagged with fluorescent markers to track and identify where mRNAs are present; how much of these mRNAs were present, whether they were associated with ribosomes needed for translation, and whether these mRNAs were bound to FMRP in different regions (i.e., dendrites and cell bodies). In another set of experiments, Hale et al. further compared wild type mice with a functional FMRP protein to mice without FMRP to identify dysregulated mRNAs relevant to FXS (Figure 1).

This revealed that FMRP regulates distinct populations of mRNAs in the dendrites and the cell bodies. In the dendrites, FMRP predominantly coordinates the translation of synaptic proteins, which can affect how a neuron responds to signals and may alter the strength of the synapses. In the cell bodies, FMRP controls the translation of proteins that are subsequently trafficked to the cell nucleus, including proteins that regulate the activity of genes. This is consistent with previous studies showing that FMRP can regulate the expression of both nuclear and synaptic proteins (Korb et al., 2017; Richter et al., 2015). However, it was not known that FMRP regulates these two disparate classes of mRNAs in a compartment specific manner.

Hale et al. further demonstrate that FMRP preferentially binds to mRNA isoforms, which encode for the same protein, but have different, untranslated regions at the end of the transcript involved in translational regulation. In connection with FMRP, the isoforms may influence the stability or the translation of mRNAs within specific parts of the neuron, but it remains to be seen how exactly FMRP regulates the selective translation of distinct mRNA isoforms in a sequence-specific manner (Tushev et al., 2018). Contrary to other studies, no specific role for RNA sequence motifs or secondary structures (which would help RNA binding proteins to recognize specific mRNAs) was found (Ascano et al., 2012; Goering et al., 2020).

Moreover, the study of Hale et al. indicates that FMRP does not appear to be necessary for localizing mRNAs to the dendrites, as the mRNAs found there were the same in mice both with and without FMRP. This is consistent with an earlier report (Steward et al., 1998) and suggests that other RNA binding proteins may be involved in transporting mRNAs. More research is needed to better understand the mechanisms of FMRP selectivity and how its targets are directly transported.

Consistent with previous research, a main conclusion from the work of Hale et al. is that the lack of FMRP in mice models for FXS leads to a regulatory loss of protein production in different neuronal compartments, which affects synaptic plasticity, learning and memory (Figure 1). The large datasets generated by Hale et al., and their innovative technique of tracking mRNAs (and their activity) in neurons, have revealed that these cells employ transcriptional and post-transcriptional mechanisms to differentially regulate protein synthesis throughout the neuron.

Likely, hippocampal neurons involved in memory formation are constantly undergoing changes in gene expression, during which transcripts need to properly localize, some restricted to the cell body and others shipped out to the synapses. This insight may help pave new ways for future studies to identify potential roles for some of these dysregulated mRNAs in FXS and other conditions caused by an altered expression of RNA binding proteins.
